# Relationship between C - Reactive Protein and Stroke: A Large Prospective Community Based Study

**DOI:** 10.1371/journal.pone.0107017

**Published:** 2014-09-05

**Authors:** Yanfang Liu, Jing Wang, Liqun Zhang, Chunxue Wang, Jianwei Wu, Yong Zhou, Xiang Gao, Anxin Wang, Shouling Wu, Xingquan Zhao

**Affiliations:** 1 Department of Neurology, Beijing Tiantan Hospital, Capital Medical University, Beijing, China; 2 Neurology Department, St George’s Hospital, London, United Kingdom; 3 Channing Laboratory, Department of Medicine, Brigham and Women’s Hospital, and Harvard Medical School, Boston, MA, United States of America; 4 Department of Nutrition, Harvard University School of Public Health, Boston, MA, United States of America; 5 Department of Cardiology, Kailuan Hospital, Hebei United University, Tangshan, China; Shanghai Institute of Hypertension, China

## Abstract

**Objective:**

Previous studies have suggested that C-reactive protein (CRP) was associated with risk of stroke. There were few studies in Asian population, or on stroke subtypes other than ischemic stroke. We thus investigated the relationship between CRP and the risks of all stroke and its subtypes in a Chinese adult population.

**Methods:**

In the current study, we included 90,517 Chinese adults free of stroke and myocardial infarction at baseline (June 2006 to October 2007) in analyses. Strokes were classified as ischemic stroke (IS), intracranial heamorrhage (ICH) and subarachnoid heamorrhage (SAH). High-sensitivity CRP (hs-CRP) were categorized into three groups: <1 mg/L, 1 to 3 mg/L, and >3 mg/L. Cox proportional hazards regression was used to calculate the association between hs-CRP concentrations and all stroke, as well as its subtypes.

**Results:**

During a median follow-up time of 49 months, we documented 1,472 incident stroke cases. Of which 1,049 (71.3%) were IS, 383 (26.0%) were ICH, and 40 (2.7%) were SAH. After multivariate adjustment, hs-CRP concentrations ≥1 mg/L were associated with increased risks of all stroke (hs-CRP 1–3 mg/L: hazard ratio (HR) 1.17, 95% confidential interval (CI) 1.03–1.33; hs-CRP>3 mg/L: HR 1.25, 95% CI 1.07–1.46) and IS (hs-CRP 1–3 mg/L: HR 1.17, 95% CI 1.01–1.36; hs-CRP>3 mg/L: HR 1.33, 95% CI 1.11–1.60), but not with ICH and SAH. Subgroup analyses showed that higher hs-CRP concentration was more prone to be a risk factor for all stroke and IS in non-fatal stroke, male and hypertensive participants.

**Conclusion:**

We found that higher hs-CRP concentrations were associated with a higher risk of IS, particularly for non-fatal stroke, male and hypertensive subjects. In contrast, we did not observe significant associations between hs-CRP and ICH/SAH.

## Introduction

According to the Global Burden of Disease Study 2010, stroke was the third leading cause of disability-adjusted life years (DALYs) worldwide [1]. The same study showed that in China, cardiovascular diseases (stroke and ischemic heart disease) were the leading cause of DALYs, and stroke was the leading cause of death [2]. Atherosclerosis is the most common cause of stroke. The development of atherosclerosis was not only associated with dyslipidemia [3], but also closely associated with inflammation. The later was linked to the complexity and instability of plaques [4]. C-reactive protein (CRP), an indicator of systemic inflammation, may predict the burden of atherosclerosis [5], [6].

Although many studies showed that CRP was associated with increased risk of stroke [7]–[10], others did not find the significant association [11], [12]. Most of these studies with positive findings focused on ischemic stroke. In addition, most of these studies were in Europe and America with majority of non-Asian population. It has been shown that Asians tend to have higher concentrations of CRP than European whites [13]. To our knowledge there is no large scale study on CRP as a risk factor of stroke or its subtypes in Chinese. We therefore conducted a large prospective study to investigate the relationship between CRP concentrations and stroke and its subtypes, i.e. ischemic stroke (IS), hemorrhagic stroke (ICH) and subarachnoid heamorrhage (SAH) using samples from Kailuan Study.

## Methods

### Study Design and Population

The Kailuan study was a longitudinal, ongoing community health study based on Kailuan community in Tangshan city, which is a large and littoral modern city located in the southeast of Beijing. From June 2006 to October 2007, all employees (including the retired) in the community (155,418) were invited to participate the study, and 65.3% of them agreed. After screening against the inclusion criteria (1) aged 18 years or older; (2) providing written informed consent; and (3) updating their health status every year and biennial measurements of the below parameters according to the follow-up protocol, 101,510 participants (81,110 men and 20,400 women, aged 18–98 years old) were recruited into the Kailuan study. All participants underwent a questionnaire survey, clinical examination, and laboratory testing, which were conducted in the 11 hospitals in Kailuan community, following standard protocols described previously [14].

In this study, we excluded subjects who had a history of myocardial infarction (MI) (1,112), stroke (2,355), or both (203) at baseline. Because hs-CRP concentrations >10.0 mg/L suggesting an acute inflammation, we also excluded participants with hs-CRP concentrations >10.0 mg/L (7,323), leaving 90,517 subjects included in the analyses. This study was performed according to the guidelines of Helsinki Declaration and was approved by the Ethics Committees of Kailuan General Hospital, Beijing Chaoyang Hospital and Beijing Tiantan Hospital, as a collaborative project.

### Assessment of Stroke and stroke subtypes

The primary outcome was the first incidence of stroke, either nonfatal or fatal. The diagnosis of stroke was made according to World Health Organization (WHO) criteria [15] confirmed with brain computed tomography (CT) or magnetic resonance (MR), and classified into one of the three subtypes: IS, ICH, or SAH, following unified protocol. A nonfatal stroke was defined as a focal neurological deficit of sudden onset and vascular mechanism that lasted >24 hours. Fatal stroke were the deaths caused by a confirmed cerebrovascular mechanism.

The stroke history of all participants was recorded biennially at clinic visits or telephone interviews from the baseline survey through December 31, 2010, or to the date of death or loss to follow-up. The results were confirmed by hospital discharge summaries or death certificates.

### Assessment of Variables

Information on demographic variables (e.g. age, sex), smoking and alcohol habit, and past medical history were collected via questionnaires administered by the research doctors at the baseline interview. Hypertension was defined by the presence of any of the following: a history of hypertension, using antihypertensive treatment, a systolic blood pressure ≥140 mmHg, or a diastolic pressure ≥90 mmHg. Diabetes mellitus was diagnosed by the presence of any of the following: a history of diabetes mellitus, currently treated with insulin or oral hypoglycemic agents, or the fasting blood glucose level was ≥7.0 mmol/L. Hyperlipidemia was defined by the presence of any of the following: a history of hyperlipidemia, current use of cholesterol lowering agents, or the total cholesterol level ≥5.17 mmol/L or triglyceride ≥1.7 mmol/L.

Body weight, height and blood pressure (Bp) were measured during the baseline interview, and body mass index (BMI) was calculated. Bp was the average of at least two readings at rest.

Blood samples were obtained after an overnight fast in EDTA tubes at the baseline interview. Fasting blood glucose was measured with the hexokinase/glucose-6-phosphate dehydrogenase method. Cholesterol and triglyceride were measured enzymatically (Mind Bioengineering Co. Ltd, Shanghai, China). High-sensitivity (Hs)-CRP was measured by high-sensitivity nephelometry assay (Cias Latex CRP-H, Kanto Chemical Co. Inc, Tokyo, Japan). All blood variables were measured using an auto-analyzer (Hitachi 747; Hitachi, Tokyo, Japan) at the central laboratory of the Kailuan General Hospital. According to the guideline from the centers for Disease Control and Prevention and the American Heart Association, hs-CRP concentrations were categorized into three groups: hs-CRP<1 mg/L, hs-CRP 1 to 3 mg/L, and hs-CRP>3 mg/L [16].

### Statistical Analysis

Statistical analyses were performed using SAS software, version 9.1 (SAS Institute, Cary, North Carolina, USA). As all the continuous variables were in skewed distribution, median were used for analysis, and compared using analysis of variance (ANOVA). Hs-CRP was log-transformed as a continuous variable to fit a less skewed distribution. Categorical variables were described by percentages, and compared using Chi-Square tests. Direct standardization method was used to calculate the age- and sex-standardized incidence rates according the National population census in 2010. Hazard ratios (HRs) and 95% confidence intervals (CI) of stroke and its subtypes were estimated according to different hs-CRP concentrations and 1-SD increment of log-transformed hs-CRP (antilog of SD = 3.0 mg/L) with Cox proportional hazards regression models [17]. Adjustment was made for age, sex, hypertension, diabetes, dyslipidemia and for SBP, DBP, BMI, TC, TG, LDL, GLU, current smoking, alcohol intake, and antihypertensive treatment, lipid-lowering treatment, hypoglycemic treatment were also conducted. The Kaplan-Meier curves for cumulative stroke and its subtypes were plotted according to hs-CRP concentrations and compared with the log-rank test. Receiver Operating Characteristic (ROC) analysis was used to determinate the cut-off-points of hs-CRP concentrations for the prediction of all stroke and stroke subtypes, as well as their predicted efficiency. All statistical tests were 2-sided, and the significant level was set at 0.05.

## Results

Baseline characteristics according to hs-CRP concentrations were presented in Table 1. Male subjects were in higher proportion (79.5%) in the study. Subjects in the groups of higher hs-CRP concentrations (≥1 mg/L) were older. High hs-CRP concentrations (≥1 mg/L) were associated with higher prevalence of traditional stroke risk factors, including hypertension (both systolic and diastolic), diabetes, dyslipidemia, high BMI, and elevated concentrations of fasting glucose, total cholesterol (TC), triglycerides (TG) and low-density lipoprotein cholesterol (LDL). Interestingly, of the three groups, the concentrations of TC, TG, LDL and the prevalence of dyslipidemia, current smoking/alcohol intake, and taking antihypertensive/hypoglycemic drug therapy were the highest in the hs-CRP of 1 to 3 mg/L group.

**Table 1:** Baseline characteristics according the hs-CRP groups.

	hs-CRP				
	Total	<1 mg/L	1–3 mg/L	>3 mg/L	P value
Number	90517	52388(57.9)	23547(26.0)	14582(16.1)	
Age, year	51.4(43.3–58.4)	50.1(42.4–56.6)	52.1(43.7–59.4)	54.9(48.3–63.2)	<0.001
Male, n(%)	71967(79.5)	42019(80.2)	18723(79.5)	11225(77.0)	<0.001
SBP (mmHg)	129.7(118.7–140.7)	126.0(115.3–140.0)	130.0(120.0–146.0)	130.0(120.0–149.3)	<0.001
DBP (mmHg)	80.0(78.7–90.0)	80.0(76.7–90.0)	80.7(79.3–90.0)	80.7(79.3–90.0)	<0.001
Hypertention, n(%)	38943(43.5)	20394(39.0)	11173(47.6)	7376(53.1)	<0.001
BMI, kg/m^2^	24.8(22.6–27.2)	24.4(22.2–26.6)	25.6(23.4–27.9)	25.4(23.1–27.8)	<0.001
TC, mmol/L	4.9(4.3–5.6)	4.9(4.3–5.5)	5.0(4.3–5.7)	4.9(4.3–5.6)	<0.001
TG, mmol/L	1.3(0.9–1.9)	1.2(0.9–1.8)	1.4(1.0–2.1)	1.4(1.0–2.1)	<0.001
HDL, mmol/L	1.5(1.3–1.8)	1.5(1.3–1.8)	1.5(1.3–1.7)	1.5(1.3–1.8)	<0.001
LDL, mmol/L	2.4(1.8–2.8)	2.4(1.9–2.8)	2.4(1.9–2.9)	2.2(1.3–2.8)	<0.001
hs-CRP, mg/L	0.7(0.3–1.9)	0.3(0.2–0.6)	1.6(1.2–2.1)	5.2(3.8–7.2)	<0.001
Dislipidemia, n(%)	31237(34.5)	16518(31.5)	9100(38.7)	5619(38.5)	<0.001
GLU, mmol/L	5.1(4.7–5.7)	5.1(4.7–5.6)	5.2(4.7–5.9)	5.1(4.6–5.8)	<0.001
Diabetes, n(%)	8055(9.1)	3707(7.1)	2596(11.1)	1752(13.2)	<0.001
Current smoker, n(%)	30621(34.5)	18249(34.9)	8371(35.7)	4001(30.3)	<0.001
Alcohol intake, n(%)	33372(37.5)	20093(38.6)	9052(38.6)	4227(32.0)	<0.001
Antihypertensive treatment, n(%)	8916(9.9)	3931(7.5)	3128(13.3)	1857(12.7)	<0.001
Lipid-lowering treatment, n(%)	667(0.7)	295(0.6)	221(0.9)	151(1.0)	<0.001
Hypoglycemic treatment, n(%)	1914(2.1)	874(1.7)	663(2.8)	377(2.6)	<0.001

BMI: body mass index; DBP: diastolic blood pressure; GLU: glucose; HDL: high density lipoprotein; hs-CRP: high-sensitivity C-reactive protein; LDL: low density lipoprotein; SBP: systolic blood pressure; TC: total cholesterol; TG: triglyceride.

Age, SBP, DBP, BMI, TC, TG, HDL, LDL, hs-CRP and GLU are shown in median (lower quartile, upper quartile). Numbers of each group, Male, Hypertension, Dislipidaemia, Diabetes, Current smoker, Alcohol intake, antihypertensive treatment, lipid-lowering treatment and hypoglycemic treatment are shown in number (percentage).

The total follow-up time was 362,163 person-years, with a median follow-up time of 49 months per participants. At the end of the study, we identified 1,472 new stroke cases, of which 1,049 (71.3%) were IS, 383 (26.0%) were ICH, and 40 (2.7%) were SAH. The age- and sex-standardized incidence per 1000 person-years of all stroke and stroke subtypes in this cohort according to hs-CRP concentrations were shown in Table 2. The incidence rates of IS and ICH increased with hs-CRP concentrations.

**Table 2:** The age- and sex-standardized incidence per 1000 person-years of stroke subtypes according to hs-CRP concentrations.

	hs-CRP			
	<1 mg/L	1–3 mg/L	>3 mg/L	P trend
All stroke	9.89	13.95	18.49	<0.01
IS	6.51	9.52	13.24	<0.01
ICH	3.04	4.36	4.61	0.04
SAH	0.33	0.68	0.64	0.17

hs-CRP: high-sensitivity C-reactive protein; ICH: intracranial heamorrhage; IS: ischemic stroke; SAH: subarachnoid heamorrhage.

Table 3 showed the hazard rations (HR) of all stroke and its subtypes according to serum hs-CRP concentrations using Cox proportional hazards models. In unadjusted models, elevated hs-CRP concentrations were associated with significant high risk of all stroke, IS and ICH, respectively. Adjusting for age and sex did not attenuate the trend in all stroke and IS in both groups of elevated hs-CRP groups (≥1 mg/L). After multivariate adjustment, elevated hs-CRP concentrations (≥1 mg/L) showed persistent association with increased risk of all stroke and IS. However, elevated hs-CRP (≥1 mg/L) was not significantly associated with the risk of ICH after adjusting for age and sex and multivariate adjustment (Table 3). There was no significant association between hs-CRP concentrations and the risk of SAH.

**Table 3:** Hazard ratios (HR) and 95% confidence intervals (95% CI) of all stroke and stroke subtypes according to serum hs-CRP concentrations.

		hs-CRP			
		<1 mg/L	1–3 mg/L	>3 mg/L	1SD increment of log hs-CRP
All stroke	N(%)	652(1.24)	440(1.87)	380(2.61)	
	Crude Model	1.00(Reference)	1.50(1.33–1.69)**	1.80(1.56–2.09)**	1.32(1.24–1.40)**
	Model 1	1.00(Reference)	1.32(1.17–1.49)**	1.45(1.24–1.68)**	1.18(1.11–1.26)**
	Model 2	1.00(Reference)	1.17(1.03–1.33)*	1.25(1.07–1.46)*	1.09(1.02–1.16)*
IS	N(%)	442(0.84)	315(1.34)	292(2.00)	
	Crude Model	1.00(Reference)	1.55(1.34–1.80)**	1.99(1.67–2.37)**	1.42(1.32–1.53)**
	Model 1	1.00(Reference)	1.35(1.17–1.56)**	1.58(1.32–1.88)**	1.26(1.17–1.36)**
	Model 2	1.00(Reference)	1.17(1.01–1.36)*	1.33(1.11–1.60)*	1.15(1.06–1.24)**
ICH	N(%)	192(0.37)	111(0.47)	80(0.55)	
	Crude Model	1.00(Reference)	1.35(1.07–1.71)*	1.43(1.05–1.93)*	1.11(0.99–1.24)
	Model 1	1.00(Reference)	1.22(0.96–1.55)	1.18(0.86–1.61)	1.02(0.91–1.14)
	Model 2	1.00(Reference)	1.15(0.90–1.47)	1.09(0.79–1.50)	0.97(0.86–1.08)
SAH	N(%)	18(0.03)	14(0.06)	8(0.05)	
	Crude Model	1.00(Reference)	1.57(0.78–3.19)	0.96(0.33–2.79)	1.32(0.90–1.93)
	Model 1	1.00(Reference)	1.49(0.73–3.04)	0.87(0.30–2.59)	1.27(0.86–1.87)
	Model 2	1.00(Reference)	1.29(0.63–2.65)	0.69(0.22–2.15)	1.14(0.76–1.70)

*P<0.05,

**P<0.001.

hs-CRP: high-sensitivity C-reactive protein; ICH: intracranial heamorrhage; IS: ischemic stroke; SAH: subarachnoid heamorrhage.

Model 1: adjusted for age and sex.

Model 2: adjusted for Model 1 and for hypertension, diabetes, dyslipidemia and for SBP, DBP, BMI, TC, TG, LDL, GLU, current smoking, alcohol intake, antihypertensive treatment, lipid-lowering treatment and hypoglycemic treatment.

To further explore the effects of hs-CRP on the outcome of stroke, and sex difference, subgroup analyses were conducted in fatal/non-fatal stroke, male/female and participants with/without hypertension (Table 4). It showed that elevated hs-CRP was a risk factor for non-fatal all stroke, and non-fatal IS. Elevated hs-CRP had significant effect on male subjects in all stroke and IS. Elevated hs-CRP was not associated with fatal stroke, and had no significant effect on female.

**Table 4:** Subgroup Analysis of hazard ratios (HR) and 95% confidence intervals (95% CI) of all stroke and stroke subtypes according to serum hs-CRP concentrations.

		hs-CRP			
		<1 mg/L	1–3 mg/L	>3 mg/L	HR for 1SD increment of log hs-CRP
Fatal stroke	All/IS/ICH/SAH, n	59/30/24/5	36/23/12/1	36/27/8/1	
All stroke	Model 2	1.00(Reference)	1.08(0.71–1.66)	1.17(0.69–1.97)	1.13(0.91–1.39)
IS	Model 2	1.00(Reference)	1.32(0.75–2.30)	1.68(0.89–3.17)	1.38(0.99–1.80)
ICH	Model 2	1.00(Reference)	0.97(0.48–1.99)	0.72(0.25–2.02)	0.92(0.66–1.28)
SAH	Model 2	1.00(Reference)	0.24(0.03–2.27)	0.12(0.00–3.50)	0.63(0.27–1.48)
Non-fatal stroke	All/IS/ICH/SAH, n	593/412/168/13	404/292/99/13	344/265/72/7	
All stroke	Model 2	1.00(Reference)	1.18(1.04–1.35)*	1.26(1.07–1.48)*	1.09(1.02–1.16)*
IS	Model 2	1.00(Reference)	1.16(1.00–1.36)	1.31(1.08–1.58)*	1.13(1.04–.23)*
ICH	Model 2	1.00(Reference)	1.18(0.91–1.53)	1.15(0.82–1.61)	0.98(0.87–1.10)
SAH	Model 2	1.00(Reference)	1.77(0.80–3.90)	0.97(0.29–3.25)	1.31(0.84–2.06)
Male	All/IS/ICH/SAH, n	588/402/171/15	387/281/96/10	326/257/64/5	
All stroke	Model 2	1.00(Reference)	1.15(1.01–1.31)*	1.25(1.06–1.48)*	1.09(1.02–1.17)*
IS	Model 2	1.00(Reference)	1.16(0.99–1.36)	1.37(1.13–1.66)*	1.07(1.04–1.11)*
ICH	Model 2	1.00(Reference)	1.13(0.87–1.46)	1.00(0.70–1.43)	1.01(0.96–1.17)
SAH	Model 2	1.00(Reference)	1.10(0.48–2.49)	0.58(0.15–2.23)	0.97(0.76–1.18)
Female	All/IS/ICH/SAH, n	64/40/21/3	53/34/15/4	54/35/16/3	
All stroke	Model 2	1.00(Reference)	1.36(0.93–2.00)	1.29(0.81–2.04)	1.06(0.88–1.29)
IS	Model 2	1.00(Reference)	1.29(0.79–2.10)	1.11(0.61–2.00)	1.08(0.84–1.32)
ICH	Model 2	1.00(Reference)	1.35(0.68–2.68)	1.66(0.76–3.63)	1.03(0.86–1.21)
SAH	Model 2	1.00(Reference)	2.51(0.53–12.03)	1.11(0.11–11.08)	1.08(0.76–1.40)
Hypertension	All/IS/ICH/SAH, n	444/303/133/8	338/241/86/11	277/208/62/7	
All stroke	Model 3	1.00(Reference)	1.19(1.03–1.38)*	1.29(1.09–1.52)*	1.11(1.03–1.19)*
IS	Model 3	1.00(Reference)	1.20(1.01–1.43)*	1.37(1.13–1.66)*	1.16(1.06–1.26)**
ICH	Model 3	1.00(Reference)	1.10(0.84–1.46)	1.06(0.76–1.47)	0.96(0.85–1.09)
SAH	Model 3	1.00(Reference)	2.23(0.89–5.61)	1.67(0.53–5.26)	1.62(0.98–2.68)
Non-hypertension	All/IS/ICH/SAH, n	208/139/59/10	102/74/25/3	103/84/18/1	
All stroke	Model 3	1.00(Reference)	1.11(0.87–1.42)	1.49(1.15–1.94)*	1.20(1.08–1.34)**
IS	Model 3	1.00(Reference)	1.15(0.86–1.53)	1.69(1.25–2.28)**	1.34(1.17–1.54)**
ICH	Model 3	1.00(Reference)	1.11(0.69–1.78)	1.14(0.65–1.98)	0.96(0.79–1.17)
SAH	Model 3	1.00(Reference)	0.72(0.19–2.65)	0.41(0.05–3.31)	0.93(0.54–1.59)

*P<0.05,

**P<0.001.

hs-CRP: high-sensitivity C-reactive protein; ICH: intracranial heamorrhage; IS: ischemic stroke; SAH: subarachnoid heamorrhage.

Model 1: adjusted for age and sex.

Model 2: adjusted for Model 1 and for hypertension, diabetes, dyslipidemia and for SBP, DBP, BMI, TC, TG, LDL, GLU, current smoking, alcohol intake, antihypertensive treatment, lipid-lowering treatment and hypoglycemic treatment.

Model 3: adjusted for Model 1 and for diabetes, dyslipidemia and for SBP, DBP, BMI, TC, TG, LDL, GLU, current smoking, alcohol intake, antihypertensive treatment, lipid-lowering treatment and hypoglycemic treatment.

Because hypertension is an independent risk factor for heamorrhagic stroke, to further explore the potential association between hs-CRP and heamorrhagic stroke, subgroup analysis was carried out based on hypertension status (yes/no) (Table 4). Hs-CRP≥1 mg/L was a risk factor for all stroke and IS in patients with hypertension, but in participants without hypertension, only hs-CRP>3 mg/L was a risk factor for all stroke and IS. Elevated hs-CRP concentrations again were not associated with the risk of ICH and SAH, regardless of hypertension status.

**Figure 1:**
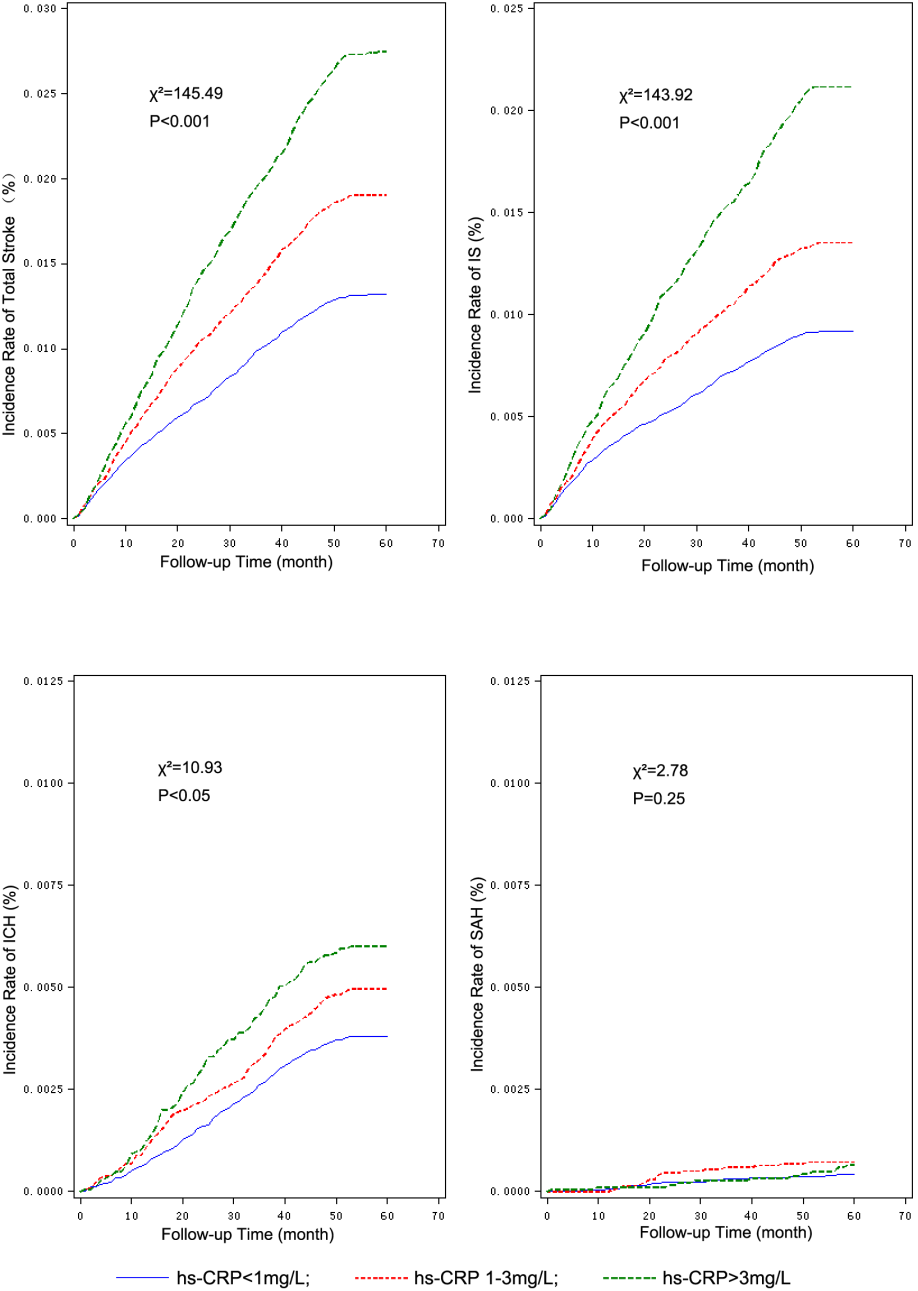
Kaplan-Meier plot for cumulative stroke and stroke subtypes according to hs-CRP concentration concentrations. hs-CRP: high-sensitivity C-reactive protein; ICH: intracranial heamorrhage; IS: ischemic stroke; SAH: subarachnoid heamorrhage. The cumulative incidence rate of all stroke, IS and ICH increased with increased concentrations of hs-CRP. There was no significant difference in the incidence of SAH in each hs-CRP groups.

The Kaplan-Meier plot of stroke and stroke subtypes according to the concentrations of hs-CRP showed the cumulative incidence rates of all stroke, IS and ICH were associated with elevated hs-CRP concentrations, but not with SAH (Figure 1). Further ROC analysis showed the cut-off-points of hs-CRP concentrations for all stroke, IS and ICH were 1.34 mg/L, 1.01 mg/L and 0.84 mg/L, respectively, and the areas under ROC curve were 0.592, 0.611 and 0.537, correspondingly.

## Discussion

Previous studies showed that CRP concentrations were modestly associated with the risk of IS [8], [10], [18] and transient ischemic attack (TIA) [10] in middle-aged [18] and elderly individuals [8], [10]. Most of these were American studies, and focused on IS. In a Japanese retrospective study, elevated CRP concentrations were associated with higher incidence of all stroke and IS, but not heamorrhagic stroke [17]. There was also a study showed high CRP level was a strong risk factor for fatal stroke [19]. Based on these studies, we conducted this community based, prospective study to explore the relationship between CRP concentrations and the incidence of stroke and its subtypes in Chinese. In this large study of 90,517 Chinese, we confirmed that elevated hs-CRP concentrations were significantly associated with increased risk of all stroke and IS, but not ICH and SAH. The higher hs-CRP concentrations, the higher incidence rates of all stroke, IS and ICH, but not that of SAH.

The pathophysiological mechanism of the association between elevated CRP concentrations and the risk of stroke is yet fully elucidated. CRP is an indicator of inflammation, both acute and chronic systemic inflammation [20]. Inflammation may promote thrombosis and vascular injury, which are responsible for most cardiovascular diseases [21]. Studies suggested that inflammation may affect the composition, morphology and stability of atherosclerotic plaque [4], [5]. An autopsy study demonstrated the existence of CRP in the atherosclerotic coronary arteries but not in the normal ones; and the intensity of CRP staining was correlated with the relative intimal thickness [22]. CRP may also promote thrombotic events by inducing monocytes to express tissue factor, a potent procoagulant [23]. Because large-artery atherosclerosis is the most common cause of IS [24], our clinical findings are in line with those studies [7]–[10], [17]–[19], showing that the elevated CRP concentrations are associated with increased risk of IS.

Research on the relationship between CRP and ICH was scarce, with limited data suggesting that CRP concentrations are not associated with ICH [17], [25], [26]. However, this study demonstrated, at least a trend, that with the increase of hs-CRP concentrations, the incidence of ICH increased. There were studies showed that CRP concentrations were associated with white matter lesions, suggesting inflammatory process involvement in the pathogenesis of cerebral small vessel disease [27]. It is known that ICH can be due to the rupture of small vessels from lipohyalinosis secondary to hypertension [28], or by amyloid angiopathy [29]. The relationship between CRP and ICH was further demonstrated by a study showing hs-CRP concentrations were associated with cerebral microbleeds, in both lobar and deep locations [30]. Cerebral microbleeds were strongly associated with the occurrence of ICH [31]. Thus, the finding in our study was not completely unexpected, although the results from multivariant adjustment suggesting elevated hs-CRP may not be an independent risk factor for ICH, or it may be only associated with certain subtype of ICH that associated with inflammation. This is in agreement with previous studies.

The results of the relationship between hs-CRP and SAH were similar to previous studies [26], [32] in which hs-CRP concentrations were not associated with the SAH risk. This might be due to the main risk factors for SAH are current smoking, hypertension and alcohol intake [33], and atherosclerosis plays a weak role in intracranial aneurysm [34]. It might also be possible that the incidence of SAH is very low, nearly 10.5 per 100,000 person years [35], which made this study under-powered to detect the significance.

The current study is a large-scale community based study, with a good number of incident stroke cases. There are also some methodological strengths, such as prospective design, and detailed follow-up and data collection. However, this study had some limitations. First, during the follow-up, some participants might start or withdraw lipid-lowering treatment, which might affect CRP concentrations and inflammation [36]. We were unable to adjust for these factors. However, at the baseline, only <1% participants took lipid-lowering medicine in this cohort, and even fewer participants changed medicine during the study. Thus the impact of the usage of lipid-lowering agents on the results can be neglected. Second, because of the industrial nature of Kailuan Community, there was an imbalance in gender distribution, more men than women. However, the incidence rates of stroke subtypes were similar in Chinese men and women [37], thus the influence of imbalance in gender distribution on the results would be minimal.

## Conclusion

We observed that elevated hs-CRP concentrations were positively associated with the risk of ischemic stroke, particularly for non-fatal stroke, male and hypertensive subjects, but not of intracranial hemorrhage and subarachnoid heamorrhage. Based on this large population study, CRP concentrations can be used as a clinical screen tool to identify individuals with higher risk of ischemic stroke in Chinese population.

## References

[1] Murray CJ, Vos T, Lozano R, Naghavi M, Flaxman AD, et al (2012) Disability-adjusted life years (DALYs) for 291 diseases and injuries in 21 regions, 1990–2010: a systematic analysis for the Global Burden of Disease Study 2010. Lancet 380: 2197–2223. 23245608 10.1016/S0140-6736(12)61689-4

[2] Yang G, Wang Y, Zeng Y, Gao GF, Liang X, et al (2013) Rapid health transition in China, 1990–2010: findings from the Global Burden of Disease Study 2010. The Lancet 381: 1987–2015. 10.1016/S0140-6736(13)61097-1 PMC7159289 23746901

[3] Paramsothy P, Knopp RH, Bertoni AG, Blumenthal RS, Wasserman BA, et al (2010) Association of combinations of lipid parameters with carotid intima-media thickness and coronary artery calcium in the MESA (Multi-Ethnic Study of Atherosclerosis). J Am Coll Cardiol 56: 1034–1041. 20846602 10.1016/j.jacc.2010.01.073

[4] Lombardo A, Biasucci LM, Lanza GA, Coli S, Silvestri P, et al (2004) Inflammation as a possible link between coronary and carotid plaque instability. Circulation 109: 3158–3163. 15184282 10.1161/01.CIR.0000130786.28008.56

[5] Khera A, de Lemos JA, Peshock RM, Lo HS, Stanek HG, et al (2006) Relationship between C-reactive protein and subclinical atherosclerosis: the Dallas Heart Study. Circulation 113: 38–43. 16380546 10.1161/CIRCULATIONAHA.105.575241

[6] Tzoulaki I, Murray GD, Lee AJ, Rumley A, Lowe GD, et al (2005) C-reactive protein, interleukin-6, and soluble adhesion molecules as predictors of progressive peripheral atherosclerosis in the general population: Edinburgh Artery Study. Circulation 112: 976–983. 16087797 10.1161/CIRCULATIONAHA.104.513085

[7] Di Napoli M, Papa F, Bocola V (2001) C-Reactive Protein in Ischemic Stroke: An Independent Prognostic Factor. Stroke 32: 917–924. 11283392 10.1161/01.str.32.4.917

[8] Cao JJ, Thach C, Manolio TA, Psaty BM, Kuller LH, et al (2003) C-reactive protein, carotid intima-media thickness, and incidence of ischemic stroke in the elderly: the Cardiovascular Health Study. Circulation 108: 166–170. 12821545 10.1161/01.CIR.0000079160.07364.6A

[9] Curb JD, Abbott RD, Rodriguez BL, Sakkinen P, Popper JS, et al (2003) C-reactive protein and the future risk of thromboembolic stroke in healthy men. Circulation 107: 2016–2020. 12681999 10.1161/01.CIR.0000065228.20100.F7

[10] Rost NS, Wolf PA, Kase CS, Kelly-Hayes M, Silbershatz H, et al (2001) Plasma Concentration of C-Reactive Protein and Risk of Ischemic Stroke and Transient Ischemic Attack: The Framingham Study. Stroke 32: 2575–2579. 11692019 10.1161/hs1101.098151

[11] Wilson PW, Nam BH, Pencina M, D’Agostino RB Sr, Benjamin EJ, et al (2005) C-reactive protein and risk of cardiovascular disease in men and women from the Framingham Heart Study. Arch Intern Med 165: 2473–2478. 16314543 10.1001/archinte.165.21.2473

[12] Bos MJ, Schipper CM, Koudstaal PJ, Witteman JC, Hofman A, et al (2006) High serum C-reactive protein level is not an independent predictor for stroke: the Rotterdam Study. Circulation 114: 1591–1598. 17015791 10.1161/CIRCULATIONAHA.106.619833

[13] Chambers JC, Eda S, Bassett P, Karim Y, Thompson SG, et al (2001) C-Reactive Protein, Insulin Resistance, Central Obesity, and Coronary Heart Disease Risk in Indian Asians From the United Kingdom Compared With European Whites. Circulation 104: 145–150. 11447077 10.1161/01.cir.104.2.145

[14] Wu S, Li Y, Jin C, Yang P, Li D, et al (2012) Intra-individual variability of high-sensitivity C-reactive protein in Chinese general population. Int J Cardiol 157: 75–79. 21215477 10.1016/j.ijcard.2010.12.019

[15] WHO Task Force on Stroke, other Cerebrovascular Disorders (1989) Stroke–1989. Recommendations on stroke prevention, diagnosis, and therapy. Report of the WHO Task Force on Stroke and other Cerebrovascular Disorders. Stroke 20: 1407–1431. 2799873 10.1161/01.str.20.10.1407

[16] Pearson TA (2003) Markers of Inflammation and Cardiovascular Disease: Application to Clinical and Public Health Practice: A Statement for Healthcare Professionals From the Centers for Disease Control and Prevention and the American Heart Association. Circulation 107: 499–511. 12551878 10.1161/01.cir.0000052939.59093.45

[17] Chei CL, Yamagishi K, Kitamura A, Kiyama M, Imano H, et al (2011) C-reactive protein levels and risk of stroke and its subtype in Japanese: The Circulatory Risk in Communities Study (CIRCS). Atherosclerosis 217: 187–193. 21444086 10.1016/j.atherosclerosis.2011.03.001

[18] Ballantyne CM, Hoogeveen RC, Bang H, Coresh J, Folsom AR, et al (2005) Lipoprotein-associated phospholipase A2, high-sensitivity C-reactive protein, and risk for incident ischemic stroke in middle-aged men and women in the Atherosclerosis Risk in Communities (ARIC) study. Arch Intern Med 165: 2479–2484. 16314544 10.1001/archinte.165.21.2479

[19] Gussekloo J, Schaap MCL, Frolich M, Blauw GJ, Westendorp RGJ (2000) C-Reactive Protein Is a Strong but Nonspecific Risk Factor of Fatal Stroke in Elderly Persons. Arterioscler Thromb Vasc Biol 20: 1047–1051. 10764671 10.1161/01.atv.20.4.1047

[20] Gabay C, Kushner I (1999) Acute-phase proteins and other systemic responses to inflammation. N Engl J Med 340: 448–454. 9971870 10.1056/NEJM199902113400607

[21] Libby P (2002) Inflammation in atherosclerosis. Nature 420: 868–874. 12490960 10.1038/nature01323

[22] Zhang YX, Cliff WJ, Schoefl GI, Higgins G (1999) Coronary C-reactive protein distribution: its relation to development of atherosclerosis. Atherosclerosis 145: 375–379. 10488966 10.1016/s0021-9150(99)00105-7

[23] Cermak J, Key NS, Bach RR, Balla J, Jacob HS, et al (1993) C-reactive protein induces human peripheral blood monocytes to synthesize tissue factor. Blood 82: 513–520. 8329706

[24] Adams HP, Bendixen BH, Kappelle LJ, Biller J, Love BB, et al (1993) Classification of subtype of acute ischemic stroke. Definitions for use in a multicenter clinical trial. TOAST. Trial of Org 10172 in Acute Stroke Treatment. Stroke 24: 35–41. 7678184 10.1161/01.str.24.1.35

[25] Welsh P, Lowe GD, Chalmers J, Campbell DJ, Rumley A, et al (2008) Associations of proinflammatory cytokines with the risk of recurrent stroke. Stroke 39: 2226–2230. 18566306 10.1161/STROKEAHA.107.504498

[26] Wakugawa Y, Kiyohara Y, Tanizaki Y, Kubo M, Ninomiya T, et al (2006) C-reactive protein and risk of first-ever ischemic and hemorrhagic stroke in a general Japanese population: the Hisayama Study. Stroke 37: 27–32. 16306468 10.1161/01.STR.0000194958.88216.87

[27] van Dijk EJ, Prins ND, Vermeer SE, Vrooman HA, Hofman A, et al (2005) C-reactive protein and cerebral small-vessel disease: the Rotterdam Scan Study. Circulation 112: 900–905. 16061741 10.1161/CIRCULATIONAHA.104.506337

[28] Caplan LR (1992) Intracerebral haemorrhage. Lancet 339: 656–658. 1347346 10.1016/0140-6736(92)90804-c

[29] Gilbert JJ, Vinters HV (1983) Cerebral amyloid angiopathy: incidence and complications in the aging brain. I. Cerebral hemorrhage. Stroke 14: 915–923. 6658995 10.1161/01.str.14.6.915

[30] Miwa K, Tanaka M, Okazaki S, Furukado S, Sakaguchi M, et al (2011) Relations of blood inflammatory marker levels with cerebral microbleeds. Stroke 42: 3202–3206. 21868735 10.1161/STROKEAHA.111.621193

[31] Viswanathan A, Chabriat H (2006) Cerebral microhemorrhage. Stroke 37: 550–555. 16397165 10.1161/01.STR.0000199847.96188.12

[32] Badjatia N, Carpenter A, Fernandez L, Schmidt JM, Mayer SA, et al (2011) Relationship between C-reactive protein, systemic oxygen consumption, and delayed cerebral ischemia after aneurysmal subarachnoid hemorrhage. Stroke 42: 2436–2442. 21757662 10.1161/STROKEAHA.111.614685

[33] Teunissen LL, Rinkel GJ, Algra A, van Gijn J (1996) Risk factors for subarachnoid hemorrhage: a systematic review. Stroke 27: 544–549. 8610327 10.1161/01.str.27.3.544

[34] van Gijn J, Rinkel GJ (2001) Subarachnoid haemorrhage: diagnosis, causes and management. Brain 124: 249–278. 11157554 10.1093/brain/124.2.249

[35] Linn FH, Rinkel GJ, Algra A, van Gijn J (1996) Incidence of subarachnoid hemorrhage: role of region, year, and rate of computed tomography: a meta-analysis. Stroke 27: 625–629. 8614919 10.1161/01.str.27.4.625

[36] Ridker PM, Danielson E, Fonseca FA, Genest J, Gotto AM Jr, et al (2009) Reduction in C-reactive protein and LDL cholesterol and cardiovascular event rates after initiation of rosuvastatin: a prospective study of the JUPITER trial. Lancet 373: 1175–1182. 19329177 10.1016/S0140-6736(09)60447-5

[37] Zhang LF, Yang J, Hong Z, Yuan GG, Zhou BF, et al (2003) Proportion of different subtypes of stroke in China. Stroke 34: 2091–2096. 12907817 10.1161/01.STR.0000087149.42294.8C

